# Trunk Muscle Activation Patterns Differ Between Those With Low and High Back Extensor Strength During a Controlled Dynamic Task

**DOI:** 10.3389/fspor.2019.00067

**Published:** 2020-01-10

**Authors:** D. Adam Quirk, Raymond D. Trudel, Cheryl L. Hubley-Kozey

**Affiliations:** ^1^School of Biomedical Engineering, Dalhousie University, Halifax, NS, Canada; ^2^School of Physiotherapy, Dalhousie University, Halifax, NS, Canada; ^3^Physiotherapy, Department of National Defense, Halifax, NS, Canada

**Keywords:** spinal stability, electromyography (EMG), low back pain, strength, principal component analysis (PCA), motor control, biomechanics

## Abstract

It is proposed that reduced function in one of the spinal systems (active, passive, and neural) outlined by Panjabi could increase the risk of experiencing a low back injury (LBI). Also proposed is that reduced function in any one system can be compensated for by adjusting the time-varying recruitment of trunk muscles. This study addressed whether those with reduced active system function (WEAK), measured as back extensor strength, would have different trunk muscle activation patterns than those with higher function (STRONG), and secondly whether this relationship would be modified following recovery from a LBI. Sixty men participated, 30 recently recovered from LBI (rLBI, 4–12 weeks post injury) and 30 who had not had a LBI in the last year (ASYM). ASYM and rLBI participants were separated into STRONG and WEAK sub-groups if their isometric back extensor strength was above or below their group median, respectively. Trunk electromyograms from 24 muscle sites were recorded during a highly controlled horizontal transfer task. Principal component analysis captured key muscle activation patterns (amplitude and temporal); then analysis of variance models tested for strength or group*strength effects on these patterns consistent with the two main objectives. Significant strength, or group by strength effects were found for 3/4 electromyographic comparisons. In general, the WEAK group required higher activation amplitudes of abdominal and back extensor muscles, and greater temporal responsiveness of back extensor muscles only to the changing external moments than those who were STRONG. Group by strength interactions found that participants in the rLBI group had greater differences between WEAK and STRONG participants for overall muscle activation amplitudes in both abdominal and back extensor muscles. This increase in muscle activation was interpreted as compensation for lower maximum force properties whereas the increased temporal responsiveness captured a greater need to modify the agonist back extensors muscle activation patterns only in response to changes in the dynamic moments. Interactions captured that the recent experience of pain (rLBI) modified the magnitude of adjustment in muscle activation patterns potentially adapting to an increased risk of instability (painful flare) events associated with a deficit (lower strength) of the active system.

## Introduction

The osteoligamentous spine is inherently unstable, unable to return to equilibrium under even low (80–90N) compressive loads (Crisco et al., 1992). To explain how the *in vivo* spine remains stable under higher loads Panjabi proposed a theoretical model based on the premise that the tissues surrounding the spine can prevent excessive vertebral body motion, or a clinical instability event, resulting in tissue damage thought to be one cause of incapacitating pain (Panjabi, 2003). This model categorizes tissues into three functional systems; passive, active and neural (Panjabi, 2003). Regarding the risk of instability, this model has two key postulations. First, is that a deficit to any one spinal system can increase the risk of an instability event, and second, as no one system alone contributes to spinal stability, other systems can modify their function to compensate for a deficit (Panjabi, 2003). There is however a lack of empirical evidence on whether deficits in individual spinal systems change the function of the other systems.

This paper focused on the active spinal system. Animal (Brown et al., 2011; Hodges et al., 2015) and human studies (Kjaer et al., 2007; Langevin et al., 2009; D'hooge et al., 2012) show musculotendinous tissues are different in those with low back injuries (LBI) compared to healthy controls including: reduced muscle cross sectional area and increased intramuscular fat and connective tissue. While these features appear in individuals with chronic low back pain (LBP) (Steele et al., 2014a), evidence is inconclusive whether individuals in the acute phase of a LBI or those who experience recurrent LBP have similar musculotendinous changes (Goubert et al., 2016). Functionally, the active system generates forces through active muscle contractions that are transmitted to bones. Musculotendinous tissues exhibit both passive and active mechanical properties, with the influence of passive mechanical properties increasing when muscles are lengthened beyond their optimal length (Arjmand and Shirazi-Adl, 2006). Thus, for an isometric test in a neutral posture, trunk muscle strength, defined as the maximal force or moment produced under volitional effort (Larivière et al., 2002), can be predicted by knowing the size (Hultman et al., 1993; Guilhem et al., 2014) and composition (intramuscular fat content) of trunk muscles (Anderson et al., 2014) and can act as a surrogate measure of active musculotendinous system function. Consistent with the structural changes, participants with chronic LBP have reduced trunk strength (Hasue et al., 1980; Newton et al., 1993) compared to asymptomatic controls [for review see (Steele et al., 2014b)] whereas similar reductions in strength were not found in those with a history of LBP compared to pain free controls (Hultman et al., 1993; McGill et al., 2003a).

Whether changes in the active system are associated with an increased risk of instability is equivocal. A recent meta-analysis found no conclusive evidence that either the size or composition of trunk muscles is predictive of risk of future LBP (Suri et al., 2015). Earlier studies report no relationship between baseline measures of back extensor strength and the risk of future LBP (Newton et al., 1993; Lee et al., 1999) and a more recent review was inconclusive on the predictive ability of baseline measures of strength (Reenen et al., 2007). Despite this some studies have shown participants with lower back extensor strength (Cho et al., 2014) or lower extensor to flexor strength ratios (Lee et al., 1999) are at an increased risk of experiencing a low back injury in a 2 and 5 year follow up, respectively. Furthermore, strength training is shown to reduce the risk of workplace LBI (Carpenter and Nelson, 1999), with a recent meta-analysis suggesting exercise alone or in combination with education is one of the most consistent methods to prevent LBP (Steffens et al., 2016).

Collectively the above studies support that the active system plays a role in maintaining spinal stability, and yet the effect of active system deficits, characterized as reduced trunk muscle strength alone have limited influence on LBP risk. According to Panjabi's theory of spinal system compensation, a deficit in the active system, should result in adaptations to the other systems to enable an individual to complete a functional task without experiencing an instability event. The most immediate way to change the stability of the spine is to adjust the time-varying activation of the muscles (active system) by modifying the function of the neural system (neuromuscular control) (McGill et al., 2003b). Neuromuscular control can be measured by evaluating the muscle responses through electromyography (EMG). The EMG to force relationship supports that changes in the amplitude of an EMG signal are related to changes in muscle force. However, many factors contribute to this relationship and controversy exist whether this relationship is linear (Larivière et al., 2002; Brown and McGill, 2007) or curvilinear (Larivière et al., 2002). Thus, in agonist muscles, after considering activation necessary to overcome increased antagonist activation amplitudes (Larivière et al., 2002; Brown and McGill, 2007), individuals who recruit greater EMG amplitudes, normalized to maximum voluntary contractions, to perform a task likely have reduced strength (Quirk and Hubley-Kozey, 2018).

Although the EMG to force relationship is generally accepted, understanding how maximum force production relates to the EMG activation during functional tasks is less well understood, especially for the trunk musculature. Studies show that individuals who have less lower limb muscle strength require higher activation amplitudes of agonist muscles during fundamental tasks involving the knee (Mizelle et al., 2003; Takai et al., 2008) and ankle joints (Takai et al., 2008). These relationships suggest stronger individuals require less EMG activation amplitudes of agonist muscles but not whether changes in strength influence the time-varying activation of muscles during a dynamic task. Studies comparing the spatial-temporal response of trunk muscles show that making a task more challenging not only increases agonist and antagonist activation amplitudes but also increases the responsiveness, defined as the relative differential (high vs. low) in muscle activation amplitudes to changing external task demands; in this case the trunk muscle responses to changing external flexion and lateral flexion moments (Butler et al., 2010; Quirk and Hubley-Kozey, 2014). Thus, weaker individuals should also have modified temporal responses of trunk muscles compared to stronger individuals.

Finally, while trunk muscle activation may differ between stronger and weaker individuals, there is evidence that pain can independently modify how individuals recruit their trunk muscles. Inducing experimental pain increases activation amplitudes (Hodges et al., 2013) and delays muscle onset and offset (Hodges et al., 2003), ultimately modifying the spatial-temporal recruitment of trunk muscles during complex tasks (van den Hoorn et al., 2014). It is theorized that these changes persist following the resolution of pain (Hodges et al., 2013). A cross-sectional study in those suspected to have similar deficits in spinal system function (older adults vs. younger LBI), found that those with a recent experience of pain had measurable differences in trunk muscle activation patterns during a functional task than those who did not (Quirk and Hubley-Kozey, 2018). This suggests that the recent experience of pain could interact with spinal system function to alter muscle activation patterns, but direct measures of spinal system function are needed.

As part of a comprehensive investigation of all three spinal systems, the aim of this study was to probe the spinal system compensation theory by assessing the interaction between active spinal system function and neuromuscular control of the trunk muscles during a dynamic task. This experiment tested for differences in abdominal and back extensor muscle activation amplitudes and patterns during a controlled dynamic lifting task between those who produce high (STRONG) vs. low (WEAK) maximal back extensor moments as an assessment of active spinal system function. The hypothesis was that the WEAK group would recruit higher EMG activation amplitudes and have greater responsiveness to changing external moments during the task than STRONG. A secondary purpose was to determine whether the recent experience of a low back injury modified the trunk muscle activation differences between those with high and low back extensor strength.

## Materials and Methods

### Participants

Participants for this study were recruited from the Canadian Armed Forces. Asymptomatic participants volunteered by responding to on base posters and base-wide recruitment e-mails. Recovered LBI (rLBI) participants were identified if they reported a back related issue resulting in altered activities of daily living for at least 3 days (Ozguler et al., 2000) to the Canadian Forces Health Services Center. Potential participants were contacted to see if they were interested in participating in the study. For both groups a self-report questionnaire screened for the following exclusion criteria: previous abdominal or back surgery, cardiovascular, respiratory or neurological conditions that place them at risk for participating in the study. For participant in the rLBI group questions determined that their recent LBI occurred 4–12 weeks prior to their data collection, was not chronic lasting longer than 12 weeks, and not recurrent where a previous injury occurred within 12 weeks prior to their most recent episode (Delitto et al., 2012). Asymptomatic participants were screened to determine that they had not experienced an activity limiting LBP (Ozguler et al., 2000) in the last year. All rLBI were deemed recovered at the time of testing reporting minimal pain [Visual Analog Scale (VAS) <30/100 mm (Boonstra et al., 2014)], minimal disability [Roland Morris Disability (RMD) <9/24 (Roland and Morris, 1983; Stratford et al., 1998)], and resuming normal activities of daily living at the time of testing. Before testing, all participants gave written informed consent consistent with the Declaration of Helsinki, to the study protocol approved by the Institutes Research Ethics Board.

### Protocol

Participants attended two sessions. During session one all participants were screened by a registered physiotherapist (RDT) to determine whether they met the minimal pain and disability criteria and confirmed the absence of neurological conditions. During session two, pain, demographic and anthropometric data were measured. Self-reports of weekly engagement in physical activity (aerobic sessions >30 min, core/ abdominal and strength training) and if they frequently lifted objects weighing over 23 kg for their work, defined as a heavy job (Seidler et al., 2009) were recorded. Participants also filled out a Tampa Kinesiophobia (Kori et al., 1990) and Pain Catastrophizing Scale (Sullivan et al., 1995) to characterize their beliefs toward pain.

**Figure 1:**
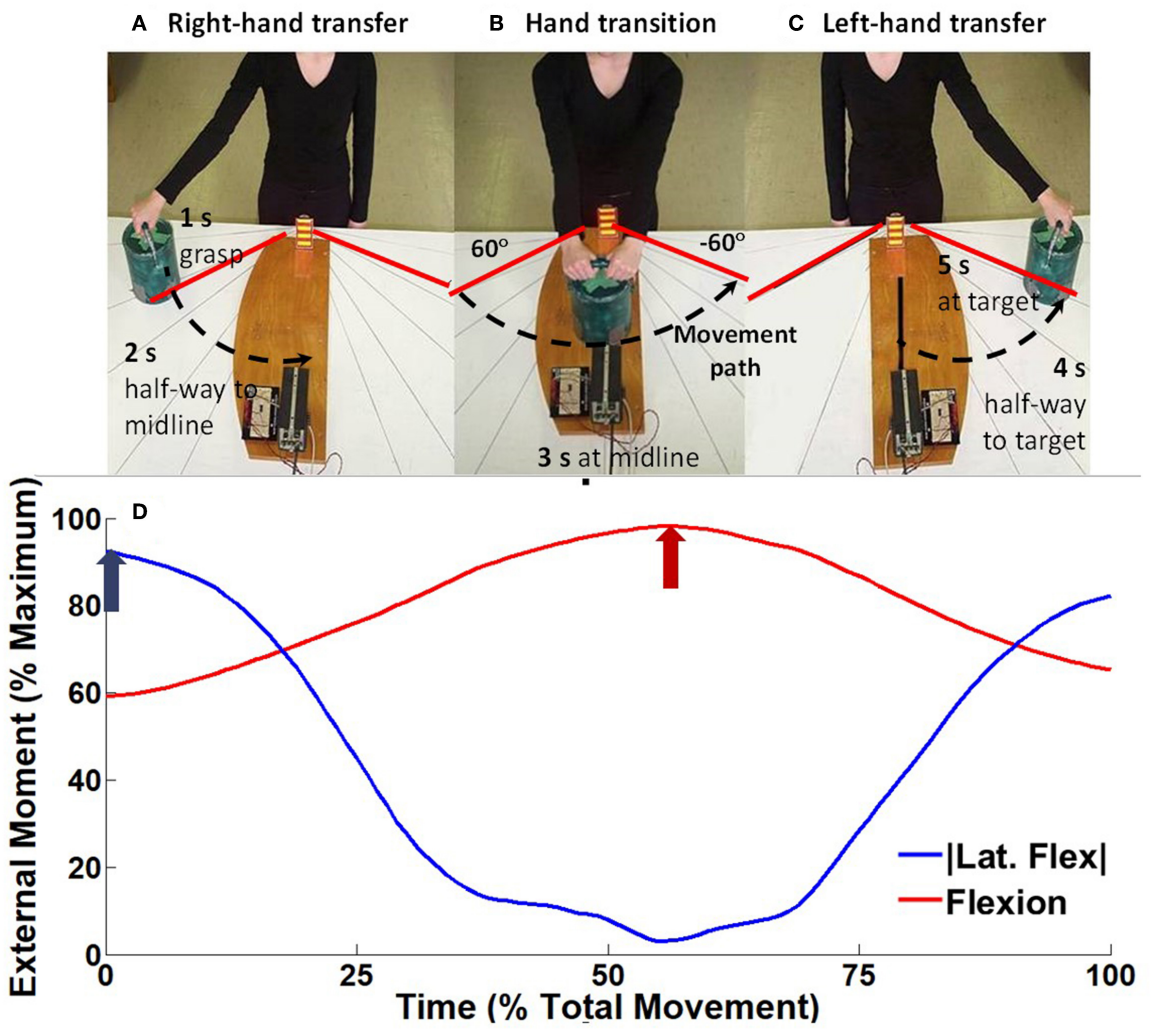
Experimental setup for the horizontal transfer task, modified from Hubley-Kozey et al. (2012). To a 5 s external count, participants would on “1” **(A)** lift a 3 kg mass orientated 60° to the right of the midline of their body with their right hand (right hand transfer RHT), on “3” **(B)** transferring the mass between hands at the midline of their body (hand transfer HT), and on “5” **(C)** lower the mass 60° to the left of their midline (left hand transfer LHT). As the participants transfer the mass from right to left **(A→C)** the ensemble average external moment generated around the spine changed from right lateral flexion moment, to a maximum flexion moment, toward a left lateral flexion moment **(D)**. For each trial the peak lateral flexion moment (blue arrow) and flexion moment (red arrow) were measured.

Participants were prepared for surface EMG (Section Surface Electromyography) and three dimensional motion capture (Section Kinematic and Kinetic Analysis) data collections, then they performed three trials of a highly controlled standardized right-to-left horizontal transfer task previously described (Butler et al., 2010) (Figure 1). Briefly, to a 5 s count, participants were instructed to on “1” lift a 3 kg mass orientated 60° to the right of the midline of their body with their right hand, on “3” transferring the mass to their left hand at the midline of their body, and then on “5” lower the mass 60° to the left of their midline. Mass lift and lower were recorded using a pressure sensor located at bottom of the mass, and hand transition was determined using an optoelectric switch. A height-adjustable table was used to ensure the mass was lifted from just below elbow height, and the optoelectric switch ensured the mass was lifted no higher than 5 cm. During the task, participants were instructed to “lift the mass with the arm in full extension and to minimize trunk and pelvis motion.” Consistent with our published protocol, tactile feedback was provided through light pressure on the upper thoracic spinous process using a wooden dowel adjusted while the participant was in their standing erect trunk posture (Butler et al., 2010). Participants were instructed to maintain contact to minimize trunk motion as trunk flexion would decrease the pressure and trunk extension would increase the pressure. A researcher monitored that task performance was consistent with the instructions and participants would repeat tasks if there was visual deviation from the instructions.

Participants then performed a series of maximum effort voluntary isometric contractions (MVIC) for EMG amplitude normalization including: (i) supine sit-up, (ii) side lying lateral flexion (left and right), (iii) prone back extension, and (iv) prone back extension coupled with axial rotation (right and left) (Butler et al., 2010) with each exercise repeated (two trials). Participants were provided verbal encouraged to maintain maximal effort for 3 s against non-elastic straps, with a 1 min rest between contractions to minimize fatigue. Following each contraction participants were asked to rate their effort and trials were repeated if perceived effort was below their maximum.

Following MVIC trunk strength (flexor and extensor moment production) was measured during two additional MVIC exercises. Participants were positioned in a prone or supine crook lying position with the HUMAC Norm Dynamometer (Computer Science Medicine Inc., Strongton, MA, USA) arm positioned anterior and inferior to the clavicle for trunk flexion, or superior and posterior to the spine of the scapula for trunk extension (Hasue et al., 1980), was secured around the torso using non-elasticized straps. The HUMAC centroid was positioned approximately 5 cm anterior to the posterior superior iliac spine, in line with the iliac crest, and non-elastic straps were secured to anchor the pelvis and shank. Following gravity correction, participants performed two trials with instructions and procedures consistent with the normalization tasks.

### Data Collection and Analysis

#### Surface Electromyography

**Figure 2:**
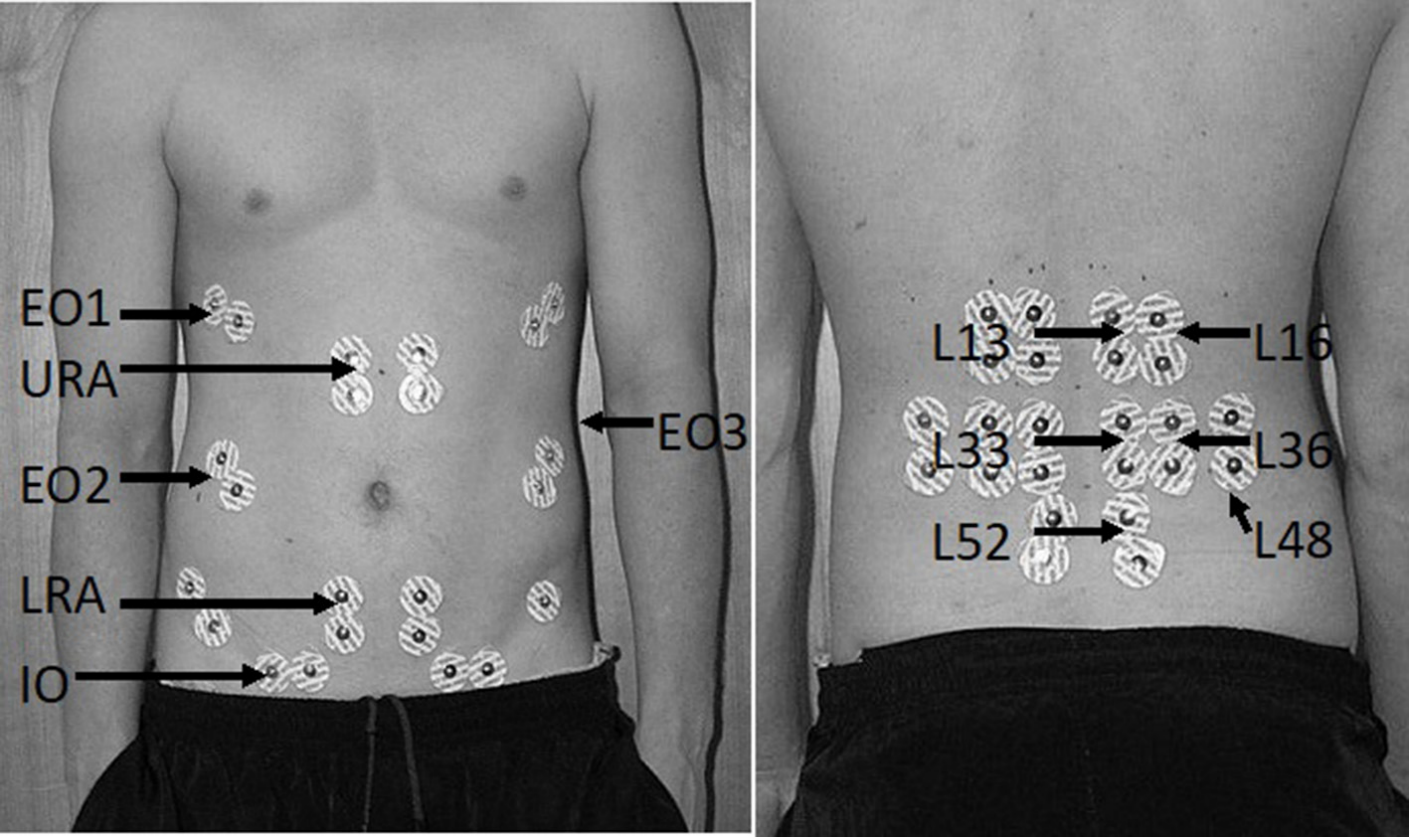
Bilateral surface electrode placement of abdominal **(left)** and back extensor sites **(right)**. Surface electrode positions are denoted by their abbreviated form including the upper (URA) and lower rectus abdominus (LRA), the anterior (EO1), lateral (EO2), and posterior external obliques (EO3), the internal obliques (IO), superficial multifidus (L52), quadratus lumborum (L48), and the iliocostalis (L16 and L36) and longissimus (L13 and L33) sites for the first and third lumbar spinous process, respectively.

Surface electrodes (Ag/AgCl, 10 mm diameter, Red dot, 3M, Maplewood, MN) were placed in a bipolar configuration (30 mm interelectrode distance) over 12 bilateral muscle sites using a standard protocol (Figure 2) (Butler et al., 2010). Abdominal sites included the upper and lower rectus abdominus (URA and LRA), the internal (IO), and external (EO1-3) oblique sites representing the anterior, lateral and posterior fibers, respectively. Back extensor sites included the superficial quadratus lumborum (L48) and multifidus (L52) along with the erector spinae at level of the 1st and 3rd lumbar spinous approximately 3 and 6 cm horizontal to the midline to capture the longissimus (L13 and L33) and iliocostalis (L33 and L36) fibers, respectively (Butler et al., 2010).

EMG signals were pre-amplified (500x) and further amplified using three AMT-8 amplifiers (band-pass 10–1,000 Hz, CMRR = 115 dB, input impedance 10 GΩ; Bortec Inc., Calgary, AB). EMG signals and event markers were digitized at 2,000 Hz using a 16-bit (± 5V) analog-to-digital board (PCI-6033E, National Instruments, Austin, TX) and Labview^TM^ (Version 2017, National Instruments). Custom Matlab (Math Works, Natick, MA) code corrected EMG signals for participant bias, electrocardiogram artifact (high pass zero-lag 30 Hz filtered) (Butler et al., 2007) and noise from electromagnetic sources [inverse fast-Fourier filtered removing frequency spikes with a power 3 times greater than their nearest frequency (±15 Hz) neighbors]. Corrected filtered data were rectified and low pass filtered at 6 Hz using a second order zero-lag Butterworth filter, to produce a linear envelope.

EMG amplitudes were normalized to the maximum average 500 ms moving window linear envelope regardless of what task evoked the MVIC (Vera-Garcia et al., 2009), and time normalized from 0 to 100% of the total task time (event markers) using a quadratic interpolation algorithm. Ensemble average waveforms were calculated from the three trials. To characterize overall neural drive the average (% MVIC) activation was calculated for the waveform for the entire task. To characterize spatial temporal characteristics, ensemble average waveforms for each participant (N) and muscle site (12) created two data matrices [(N*12) × 101] for the abdominals and back extensors separately. Each data matrix was entered into a principal component (PC) analysis model (Hubley-Kozey and Vezina, 2002). Briefly, each data matrix was transformed into a covariance matrix and underwent an eigenvector decomposition to identify eigenvectors (PCs) which explained patterns of maximum variation within the waveforms. For each EMG waveform, a coefficient (PC score) was calculated to fit the PC to the original waveform. The number of PCs extracted was determined such that the total explained variance explained by the combinations of PCs reached 90%, and that each PC explained at least 1% variance (Hubley-Kozey and Vezina, 2002).

#### Kinematic and Kinetic Analysis

Passive reflective markers were positioned on the suprasternal notch and 7th cervical spinous process along with 16 bilateral landmarks on the limbs: 5th and 2nd metacarpal, radial styloid, ulnar styloid, medial and lateral humeral epicondyle, the mid acromion clavicular joint, and the anterior superior iliac spine. Four marker rigid body clusters were affixed to the thorax and pelvis, along with bilateral clusters on the forearm and upper arm to capture motion. Following marker setup, a single standing calibration trial captured the marker position relative to the rigid bodies using six infrared emitting cameras (ProReflex 240, Qualisys^TM^, Goteborg, Sweden) sampled at 100 Hz using Qualisys Track Manager Software (Version 2.10, Qualisys^TM^, Goteborg, Sweden). For synchronization, the Labview program collecting analog-to-digital data triggered the motion capture system. Analog-to-digital pressure sensor data (event markers) were used to identify the onset (mass lift) and offset (mass lower) to determine total task time for a trial and define the window to analyze electromyographic, kinematic and kinetic data. For each participant the time to complete a trial was calculated as the difference between offset and onset, which was averaged over the three trials.

Marker data were processed in Qualisys Track Manager, to label marker's coordinates. Marker coordinate data were entered into a custom Matlab^TM^ script for quadratic interpolation of missing data points, and low pass filtered at 4 Hz using a fourth order, zero-lag Butterworth filter. Kinematic data were processed in accordance with the International Society of Biomechanics recommendation (Wu et al., 2002, 2005). Within each joint, an anatomical coordinate system was defined using bony landmarks, and joint centers as calculated using regression equations (Dumas et al., 2006). Segment angles were calculated using Euler angles in a Z, Y, X (lateral flexion, axial rotation, flexion-extension) rotation sequence. To quantify the motion during the horizontal transfer task segment angles were low pass filtered using a 1 Hz second order, zero-lag Butterworth filter (Butler et al., 2010). For each trial, the total displacement (maximum—minimum) of the torso and pelvis was calculated in lateral flexion, flexion extension, and axial rotation and averaged over the three trials for each participant.

Kinematic data were used to calculate the moments of force around the trunk using a top-down static inverse dynamics approach. Known external mass and segment mass, estimated using anthropometrically derived regression equations (Dumas et al., 2006), were inputs for the joint force and moment calculations using a system of Newton-Euler equations (Winter, 2009). An open kinetic model including joint forces and moments from distal joints were used to determine forces and moments occurring at the proximal joint. The mass of the lifted object was assumed to apply a weight vector at the middle of the 2nd and 5th metacarpal of the right-hand for the beginning of the lift, both hands during hand transition, and the left-hand following hand transition. Following this calculation, the peak lateral flexion and flexion moment was calculated for each trial and then averaged across trials for each participant (Figure 1D).

### Strength Analysis

For strength testing, gravity corrected external moments sampled from the HUMAC, were digitized at 2,000 Hz using the same analog-to-digital converter for EMG acquisition (section Surface Electromyography). Custom Matlab code filtered these data using a 6 Hz second order zero-lag low pass Butterworth filter, and converted them to moment (Nm) according to equations provided by HUMAC. The maximum isometric moment for flexors and extensors was calculated as the highest average moment over a 500 ms window which was normalized to body mass to compensate for anthropometric differences between participants (Smith et al., 1985).

### Statistical Analysis

To test whether there were differences in muscle activation amplitudes and patterns between those with higher (STRONG) or lower strength (WEAK); a median split approach was applied to the mass normalized back extensor moments, within the rLBI and asymptomatic groups. Two-way analysis of variance models (ANOVA) (group and strength) tested for differences in demographics, anthropometrics, maximum flexor and extensor moment, motion, lifting moments, and timing data. Categorical data were compared using a Chi-square test for independence. These comparisons determined if confounders should be included as co-variates when testing for muscle activation (PC) differences. Normality of PC scores was assessed using a Kolmogorov-Smirnov test, with non-normal data transformed using a Johnson transformation. PC scores were analyzed using three-factor (group, strength, and muscle) mixed model ANOVAs for the abdominals and back extensors separately. Given previous work has shown PC1 is highly correlated to the overall muscle activation amplitude (Quirk and Hubley-Kozey, 2014), pearson correlation coefficients were calculated between average EMG amplitudes and PC1 scores for each individual back and abdominal muscle site to confirm this relationship and that the average amplitude provides a physiological references for otherwise unitless PC scores. Tukey simultaneous tests compared pairwise differences when significant. Statistical analyses were performed in Minitab (version 17, State Collage, PA). Alpha was set at 0.05 and Bonferroni corrected.

To compare the relative difference between the STRONG and WEAK groups for EMG and normalized strength, the percent ratios (STRONG:WEAK) were calculated for normalized muscle strength in Nm/Kg and for EMG activation amplitudes in percent MVIC within each subgroup (rLBI and ASYM).

## Results

### Anthropometrics, Demographics, Strength, and Task Performance

Sixty-nine participants volunteered for the study and of those nine were women. Only two women were in the rLBI group. Given sex modifies muscle activation patterns (Hubley-Kozey et al., 2012) and only 2 women were in the rLBI group, the analyses were performed on the 60 men (30 rLBI reported LBI occurred 9 ± 2 weeks from the day of testing). For the STRONG and WEAK categories, the median back extensor moment threshold was 2.46 and 2.51 Nm/kg for the ASYM and rLBI, respectively. Characteristics of the separate participant groups are shown in Table 1. No group by strength interactions were significant. Significant group main effects found that the rLBI group had higher mass (*p* = 0.001), pain [VAS (*p* = 0.001)], disability [RMD (*p* = 0.003)], pain catastrophizing [PCS (*p* = 0.004)] and kinesiophobia [TSK (*p* < 0.001)] scores than ASYM. Differences between the STRONG and WEAK groups were significant for both the non-normalized (*p* < 0.008), and normalized maximum moment produced for both trunk flexors and extensors (*p* < 0.001) (Table 1).

**Table 1:** Demographics, anthropometrics, pain characteristics, self-reported physical activity, occupational loading, and trunk muscle strength.

**Strength (n)**	**STRONG (30)**		**WEAK (30)**	
Group (n)	ASYM (15)	rLBI (15)	ASYM (15)	rLBI (15)
Age (years)	35.4 (10.4)	34.6 (10.7)	34.5 (8.4)	42.1 (7.2)
Mass (kg)	78.0 (9.5)^*^	86.5 (13.8)	82.5 (14.0)^*^	91.8 (15.8)
Height (cm)	173.1 (7.6)	178.2 (6.5)	177.4 (8.1)	181.7 (7.2)
BMI (kg/m^2^)	26.0 (2.7)	27.3 (4.6)	26.2 (4.1)	27.8 (3.9)
Heavy Job [n (%)]	4 (27%)	5 (33%)	4 (27%)	5 (33%)
L. Hand [n (%)]	4 (27%)	2 (13%)	2 (13%)	1 (7%)
VAS (/100 mm)	0.9 (2.0)^*^	5.1 (8.5)	0.3 (1.0)^*^	7.1 (8.4)
PCS (/52)	6.3 (7.7)^*^	10.9 (8.1)	7.9 (6.9)^*^	16.9 (11.2)
TSK [/68 (min 17)]	30.1 (7.6)^*^	35.6 (5.2)	29.7 (6.5)^*^	37.2 (7.0)
Aerobic Training (/week)	3.7 (1.6)	3.8 (3.2)	5.1 (3.1)	2.8 (2.8)
Strength Training (/week)	2.6 (1.9)	2.9 (2.7)	2.2 (2.1)	2.1 (2.6)
Core Training (/week)	1.8 (1.6)	1.5 (1.7)	2.4 (2.2)	2.8 (2.3)
RMD (/24)	0.1 (0.3)^*^	2.4 (2.9)	0.3 (0.8)^*^	2.2 (2.1)
Norm Flexor Mo (Nm/kg)	1.8 (0.5)	1.9 (0.3)	1.5 (0.3)^†^	1.3 (0.3)^†^
Norm Ext. Mo (Nm/kg)	2.9 (0.3)	3.1 (0.4)	2.0 (0.3)^†^	2.1 (0.3)^†^

Significant differences (p < 0.05) represented by

* to indicate the difference between the ASYM and rLBI group, and

† *represent a difference between the STRONG and WEAK group. ASYM, asymptomatic; rLBI, recovered low back injury; BMI, body mass index; L. Hand, left handed; VAS, visual analog scale; PCS, pain catastrophizing scale; TSK, Tampa scale of kinesiophobia; RMD, Roland Morris Disability score; Mo, moment; Norm, normalized; Ext, extensor. The maximum attainable score for VAS, PCS, TSK, and RMD is indicated in the denominator (/Max) of column 1*.

Time to complete the task, trunk and pelvis motion, and peak external moments for the horizontal transfer task are shown in Table 2. There were no group or strength main effects or interactions (*p* > 0.05) supporting that the controlled task parameters of timing (approximately 4 s), minimizing trunk and pelvis motion and task demand of the external moment was consistent among subgroups.

**Table 2:** Horizontal transfer task performance data, timing, motion, and external moments.

**Strength (n)**	**STRONG (30)**		**WEAK (30)**	
Group (n)	ASYM (15)	rLBI (15)	ASYM (15)	rLBI (15)
Time (s)	4.3 (0.3)	4.2 (0.3)	4.2 (0.3)	4.3 (0.3)
Torso Flex/Ext (°)	3.2 (1.6)	5.1 (2.8)	4.1 (1.7)	3.8 (1.5)
Torso Lat. Flex (°)	3.2 (1.2)	2.4 (1.0)	2.7 (1.2)	2.5 (1.6)
Torso Ax Rot (°)	5.6 (2.0)	5.3 (2.8)	4.5 (2.1)	4.3 (1.8)
Pelvis Flex/Ext (°)	1.5 (1.3)	1.7 (1.0)	1.7 (0.9)	1.2 (0.8)
Pelvis Lat. Flex (°)	1.3 (0.8)	1.4 (1.0)	1.4 (0.9)	1.4 (0.9)
Pelvis Ax Rot (°)	2.5 (1.4)	2.6 (1.1)	2.4 (1.3)	2.1 (0.9)
Norm Peak Flex (Nm/kg)	0.34 (0.03)	0.33 (0.03)	0.34 (0.02)	0.32 (0.02)
Norm Peak Lat. Flex (Nm/kg)	0.15 (0.04)	0.14 (0.04)	0.15 (0.03)	0.13 (0.03)

*No significant group or strength main effects or interactions (p > 0.05). Flex, flexion; Ext, extension; Lat, lateral; Ax, axial; Rot, rotation; Norm, normalized; ASYM, asymptomatic; rLBI, recovered low back injured*.

### Trunk Muscle Activation Patterns

**Figure 3:**
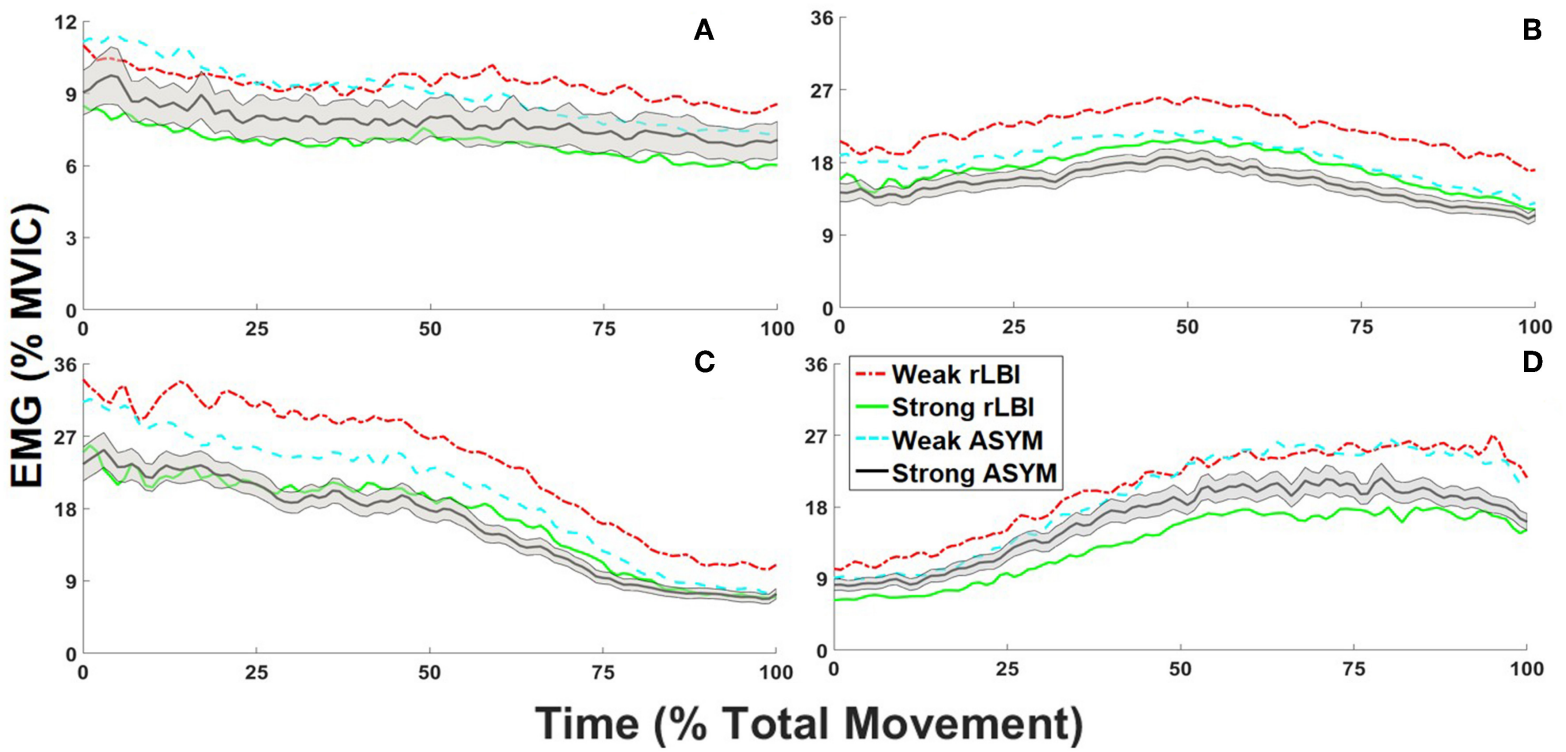
Normalized to maximum voluntary isometric contraction [%MVIC] ensemble average trunk electromyograms (EMG) for recovered low back injury [rLBI] and asymptomatic [ASYM] STRONG and WEAK subgroups. For all waveforms the standard error is depicted for the STRONG ASYM subgroup only (black line with gray shading), however standard deviations are provided in accompanying tables. For the abdominals, group by strength interaction are depicted by the contrast between **(A)** the ensemble average of right anterior external (REO1) and internal obliques [RIO] and the left middle (LEO2) and lateral external oblique sites (LEO3) for the overall amplitude (PC1). For the back extensors, group by strength interactions are depicted by **(B)** the ensemble average of both the right and left medial back extensor sites (L13, L33, and L52) for the overall amplitude (PC1) and the **(C)** left and **(D)** right lateral back extensor sites (L16 and L36) to convey temporal responsiveness (PC2).

To address the primary objective, strength main effects and interactions are first presented along with any other main effects and interactions to fully describe the data. Representative ensemble average profiles for the abdominals, and back extensors are shown in Figures 3A–D, respectively to depict group by strength interactions.

**Figure 4:**
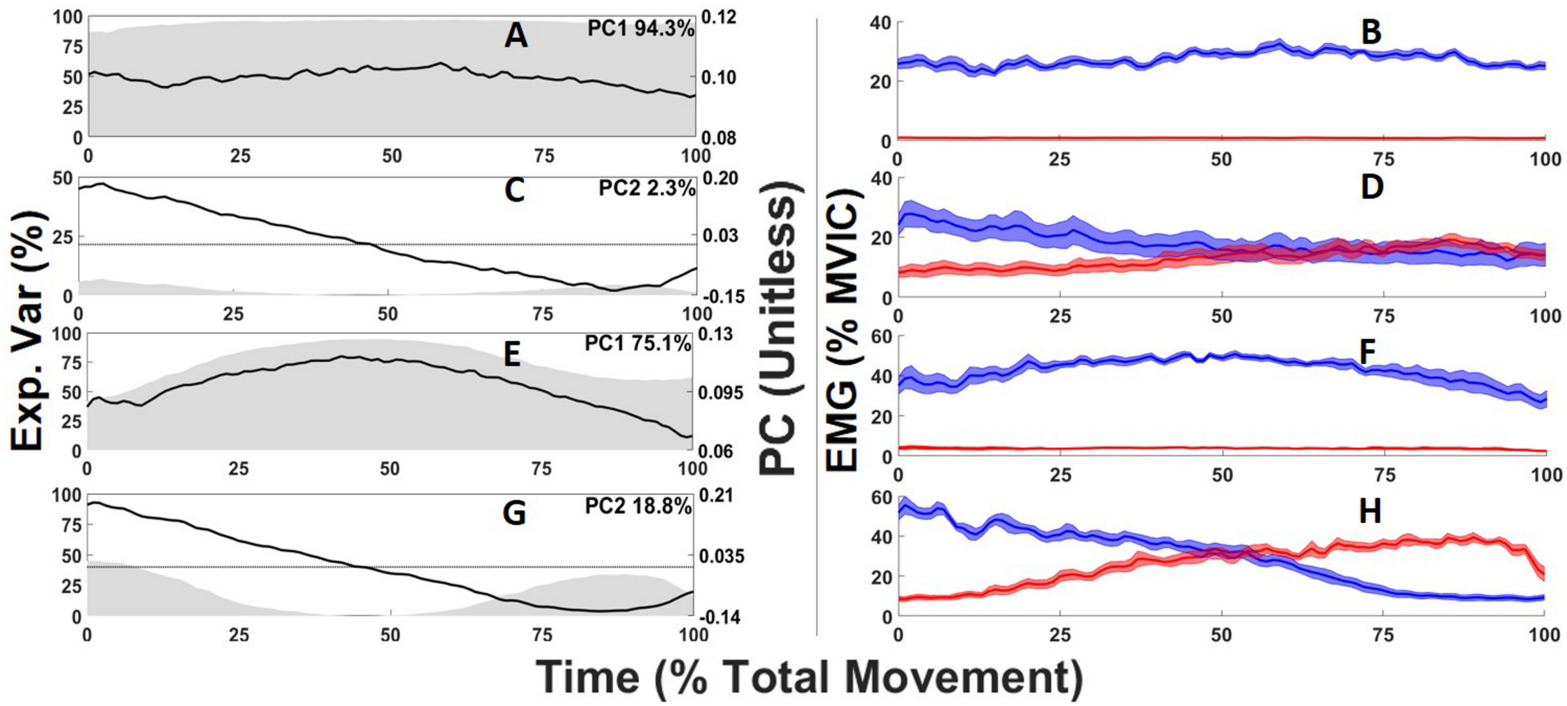
Abdominal **(A,C)** and back extensor **(E,G)** principal component (PC) waveforms (black line) where gray shading depicts the time-varying explained variance with the total variance for each PC displayed on the top right of each sub-plot. Ensemble averages electromyogram waveforms corresponding to the 5 highest PC scores (blue) and five lowest PC scores (red) for each PC waveform and their standard error (blue or red shading) are depicted in the right column **(B,D,F,H)**.

Two PC's explained 96.6% of the total abdominal muscle and 93.9% of the total back extensor muscle activation waveform variance. For both the abdominals and back extensors PC1 captured the overall waveform shape and amplitude (Figures 4A,E) where high scores captured higher activation amplitudes (Figures 4B,F). PC1 scores were highly correlated (*r* = 1) with the average amplitude of muscle sites across the entire task so the average activation amplitudes in %MVIC are also provided to give context to PCs unitless score to facilitate data interpretation. For both the abdominals and back extensors, PC2 captured a differential in periods of higher vs. lower muscle activation in response to the changing lateral flexion moment (Figures 4C,G). High scores corresponded with muscle sites having higher initial (0–25%) activation vs. lower terminal (75–100%) activation, whereas low (negative) scores corresponded to the opposite with lower initial activation (Figures 4D,H). Beyond directionality, captured by positive and negative scores, the magnitude of a PC2 score captures relative “responsiveness.” High magnitude scores convey a greater relative change (differential) in muscle activation patterns to the changing external moment whereas a low magnitude score captures that the muscle activation pattern had consistent activation occurring at the beginning and end of the task.

#### Abdominal Muscle Activation Patterns

The average amplitudes were <8% MVIC for the abdominals (Table 3), with the IO sites having the highest value but still <12% MVIC (Table 4). There was a significant group by strength interaction (*p* = 0.046) for PC1 scores where both ASYM and rLBI WEAK groups had higher activation amplitudes than their respective STRONG group (Table 3 and Figure 3A). In addition, a muscle main effect captured differences in the activation amplitudes between muscle sites showing IO sites had the highest activation, followed by EO sites with the RA sites having the lowest activation (Table 4).

**Table 3:** Strength main effects and group by strength interactions for abdominals and back extensor PC scores.

**Strength (n)**	**STRONG (30)**		**WEAK (30)**	
Group (n)	ASYM (15)	rLBI (15)	ASYM (15)	rLBI (15)
Abs. AVG (% MVIC)	6.5 ± 5.5	6.1 ± 4.5	7.0 ± 5.3	7.5 ± 6.0
Abs. PC1 (unitless)	65.5¶|| ± 55.7	61.7¶|| ± 45.5	70.5 ± 53.4	75.5 ± 60.1
Abs |PC2| (unitless)	5.5 ± 9.3			
Back AVG (% MVIC)	15.6 ± 7.6	16.2 ± 8.3	19.3 ± 8.0	22.3 ± 10.4
Back PC1 (unitless)	157.5¶|| ± 77.1	163.8¶|| ± 84.3	194.7|| ± 81.8	225.4 ± 105.7
Back |PC2| (unitless)	37.6^†^ ± 27.4		47.6 ± 30.4	

*If no main effect or interaction was identified the combined sample mean ± standard deviation is indicated in the center of the table or subgroup. Comparisons for PC2 are performed on the absolute value |PC2| scores. Significant differences (p < 0.05) for strength main effects are indicated by^†^. Significant group by strength interactions are indicated by a specific symbol to show difference relative to the: WEAK rLBI (||) and WEAK ASYM (¶) subgroup. ASYM, asymptomatic; rLBI, recovered low back injury; AVG, average activation amplitudes; % MVIC, maximum voluntary isometric contraction; PC, principal component; Abs, abdominals; Back, back extensors*.

**Table 4:** Muscle main effects and interactions for abdominal sites.

	**RLRA**	**LLRA**	**RURA**	**LURA**	**REO1**	**LEO1**	**REO2**	**LEO2**	**REO3**	**LEO3**	**RIO**	**LIO**
AVG (%MVIC)	3.7 ± 2.6	3.7 ± 2.4	3.8 ± 3.2	3.3 ± 2.7	6.9 ± 4.7	7.8 ± 6.3	6.8 ± 4.4	6.9 ± 5.5	7.5 ± 4.8	8.2 ± 4.9	11.1 ± 6.0	11.6 ± 6.4
PC1	36.8^cdef^ ± 25.9	37.1^cdef^ ± 24.3	38.5^cdef^ ± 31.7	33.5^cdef^ ± 26.9	69.6^f^ ± 47.5	78.0^f^ ± 63.8	67.9^f^ ± 44.1	69.8^f^ ± 55.2	75.7^f^ ± 48.6	82.2^f^ ± 48.8	111.4 ± 60.1	117.1 ± 65.1
PC2 ASYM	0.1^c^ ± 0.6	0.0^c^ ± 0.7	0.4^c^ ± 1.3	−1.0^c^ ± 1.4	10.3^*^± 15.5	–**13.4 ± 16.4**	−2.1^c^ ± 4.9	4.9^cf^ ± 12.1	–**5.7**^**cf**^ **± 11.8**	**14.7**^**cf**^ **± 18.1**	**3.1 ± 7.7**	–**4.0 ± 7.0**
PC2 LBI	−0.1 ± 1.1	−0.5 ± 1.4	−0.1 ± 2.0	−0.7 ± 1.8	1.6^*^± 6.2	–**6.5 ± 17.0**	−2.8 ± 4.7	0.7^f^ ± 6.4	–**6.4**^**f**^ **± 9.0**	**10.9**^**cf**^ **± 11.4**	**3.5 ± 8.5**	–**6.7 ± 11.2**

All values are mean ± standard deviation and unitless unless otherwise indicated. If only a muscle main effect exists the combined average all groups is shown, otherwise for interactions the respective group (LBI vs. ASYM) is indicated by the row title. Significant (p < 0.05) differences between muscle sites are indicated by bold lettering to show an asymmetry between left and right sites, and between muscle site differences amongst ipsilateral sites are indicated by superscript letters indicating a difference between the indicated muscle site and: (a) LRA, (b) URA, (c) EO1, (d) EO2, (e) EO3, (f) IO. Interaction effects also include the symbols

* *to indicate significant (p < 0.05) differences between the LBI and ASYM group within a specific muscle site. AVG, average activation amplitudes; % MVIC, maximum voluntary isometric contractions; PC, principal component; ASYM, asymptomatic; rLBI, recovered low back injured*.

For the abdominal PC2 scores, a group by muscle interaction (*p* = 0.004) found that the ASYM group had higher responsiveness of the right EO1 compared to the rLBI group (Table 4). This interaction also captured differences in the synergistic relationship among muscle sites where both ASYM and rLBI groups had asymmetries in the EO1, EO3, and IO. Finally, the ASYM group had more differences among ipsilateral muscle site PC2 comparisons than the rLBI (Table 4).

Given PC2 captured both the directionality (positive or negative Table 4) and magnitude (coefficient) of the responsiveness to the lateral flexion moment, data were transformed to absolute values to explore whether the magnitude of responsiveness, regardless of direction, differed between strength groups (Quirk and Hubley-Kozey, 2018) but there were no significant differences (Table 3).

#### Back Muscle Activation Patterns

For PC1, there was a group by strength interaction (*p* = 0.047) where within both the rLBI and ASYM groups, WEAK had higher activation amplitudes than STRONG (Table 3 and Figures 3B–D). This interaction also found that the WEAK rLBI subgroup had higher activation amplitudes than all other subgroups, and WEAK ASYM subgroup had higher activation than the STRONG rLBI subgroup (Table 3). Differences in muscle activation amplitudes were captured by a group by muscle interaction (*p* = 0.031) showing that there were differences in muscle synergies (Table 5). Specifically, the rLBI group had higher activation amplitudes of the L52 site between more ipsilateral sites (5/12 comparisons), than the ASYM group (3/12 comparisons) (Table 5).

**Table 5:** Muscle main effects and interactions for back extensor sites.

	**RL13**	**LL13**	**RL16**	**LL16**	**RL33**	**LL33**	**RL36**	**LL36**	**RL48**	**LL48**	**RL52**	**LL52**
AVG ASYM (%MVIC)	17.6 ± 7.0	19.2 ± 8.2	17.9 ± 6.9	19.2 ± 8.1	16.4 ± 7.5	16.7 ± 7.7	14.0 ± 6.9	15.6 ± 8.2	16.6 ± 9.7	17.0 ± 8.6	19.9 ± 8.2	18.9 ± 8.2
AVG rLBI (%MVIC)	17.3 ± 8.5	21.7 ± 10.1	16.4 ± 7.1	20.9 ± 9.3	18.2 ± 8.9	19.8 ± 10.2	15.6 ± 10.3	18.3 ± 9.9	18.4 ± 10.3	18.6 ± 11.1	22.0 ± 8.9	24.4 ± 10.0
PC1 ASYM	176.9 ± 70.6	196.8 ± 84.3	178.2 ± 69.3	195.5 ± 83.6	164.8^f^ ± 75.7	171.1 ± 79.0	139.2^f^ ± 68.8	158.9 ± 83.4	166.5^f^ ± 97.6	171.4 ± 86.7	201.6 ± 83.1	191.6 ± 8.2
PC1 rLBI	172.7^f^ ± 85.0	222.2 ± 102.8	**162.8**^**f**^ **± 70.5**	**214.0 ± 94.7**	181.8 ± 88.8	203.0 ± 103.1	155.4^f^ ± 103.0	186.8^f^ ± 100.5	183.9 ± 103.5	187.9^f^ ± 112.3	221.4 ± 88.9	247.7 ± 100.8
PC2 STRONG	**−48.4 ± 23.2**	**45.6**^†^ **± 37.4**	**−48.8 ± 27.7**	**52.5**^†^ **± 37.6**	**−45.1 ± 22.4**	**31.9 ± 25.7**	**−35.4 ± 17.4**	**36.3 ± 30.3**	**−29.1 ± 18.7**	**20.6**^**ab**^ **± 20.6**	**−32.7 ± 19.3**	**12.6**^**ab**^ **± 30.0**
PC2 WEAK	**−65.9 ± 32.4**	**57.6 ± 27.7**	**−64.4 ± 30.6**	**74.9 ± 29.4**	**−49.8 ± 32.3**	**41.8**^**b**^ **± 23.6**	**−47.4 ± 27.9**	**48.5**^**b**^ **± 28.7**	**−33.4**^**ab**^ **± 20.7**	**31.3**^**ab**^ **± 17.5**	**−36.9**^**ab**^ **± 26.1**	**12.6**^**ab**^ **± 22.2**

All values are mean ± standard deviation and unitless unless otherwise indicated. If only a muscle main effect exists the combined average of all groups is shown, otherwise for interactions the respective group (LBI vs. ASYM, STRONG vs. WEAK) is indicated by the row title. Significant (p < 0.05) differences between muscle sites are indicated by bold lettering to show an asymmetry between left and right sites, and between muscle site differences amongst ipsilateral sites are indicated by superscript letters indicating a difference between the indicated muscle site and: (a) L13 (b) L16, (c) L33, (d) L36, (e) L48, (f) L52. Interaction effects also include the symbols

† *to indicate significant (p < 0.05) differences between the STRONG and WEAK group within a specific muscle site. AVG, average activation amplitudes; % MVIC, maximum voluntary isometric contractions; PC, principal component; ASYM, asymptomatic; rLBI, recovered low back injured*.

For PC2 there was a strength by muscle interaction (*p* < 0.001) showing that WEAK was more responsive (higher PC2 scores) than STRONG for the LL16 site (Table 5). There were asymmetries between right and left sites for both strength groups. However, WEAK had more between muscle site differences than STRONG (Table 5). Transforming PC2 scores to absolute values found a strength main effect (*p* < 0.001) capturing WEAK was more responsive (higher scores) to the changing lateral flexion moment than STRONG (Table 3 and Figures 3C,D).

Percent ratios are displayed in Table 6. For the abdominals, both the EMG and strength ratio between STRONG and WEAK were greater in the rLBI than the ASYM group suggesting the elevated EMG ratio could be explained by the relative strength ratio. However, for the back extensors, while the relative strength ratios were similar for both ASYM and rLBI, EMG amplitude ratios for the rLBI group were slightly higher than ASYM (Table 6).

**Table 6:** Ratio difference between mass normalized moment (Nm/Kg) and average EMG activation amplitudes (% MVIC).

**Group**	**ASYM**	**rLBI**
Flexor Str	120%	145%
Abs EMG	117%	122%
Extensor Str	145%	148%
Back EMG	128%	137%

*Percent ratios were calculated for abdominal and back extensor strength (ratio of STRONG:WEAK) and EMG activation amplitudes in % MVIC [ratio of WEAK:STRONG] within each group (rLBI and ASYM). Nm, Newton meters; Kg, kilograms; % MVIC, maximum voluntary isometric contraction; Str, strength; Abs, abdominals; EMG, electromyography; Back, back extensor*.

## Discussion

This study explored the spinal system compensation theory by examining whether lower back extensor strength, indicative of a deficit in active spinal system function, resulted in adaptations to trunk muscle activation patterns during a dynamic task. Secondly, it examined whether the presence of a recent lower back injury that was deemed recovered modified that relationship. Fundamental was that all demographic variables and task performance variables were not different between the STRONG and WEAK groups with the only difference being muscle strength for both abdominals and back extensors.

Despite no differences in task performance, there were differences in the muscle activation amplitudes and patterns for abdominal and back extensor muscles between the STRONG and WEAK group. As hypothesized, relative to STRONG, the WEAK group had higher overall activation amplitudes of both their abdominal and back extensor sites and the back extensor sites were more responsive to the lateral flexion moment generated during task initiation and termination.

To understand whether the experience of recent LBP modified the relationship between strength and muscle activation, the group by strength interactions suggest that both groups responded to strength differences in a similar way but lower back extensor strength did have more influence on muscle activation pattern adaptations in the rLBI group. This supports that recent pain did modify these differences. These results are discussed by comparing to pertinent literature and how they relate to Panjabi's compensation theory.

### Influence of Strength on Muscle Activation Patterns

Three of the four EMG comparisons examined had a significant strength main effect or group by strength interaction. Consistent with the EMG to force relationship (Brown and McGill, 2007), WEAK recruited more motor units, reflected by higher EMG amplitudes of the primary agonist back extensor muscles (Butler et al., 2010). This finding is consistent with studies of lower limb muscles that showed for a variety of tasks (calf raise, walking, sit-to-stand, stair ascent, and descent) the maximum muscle specific strength was negatively correlated with agonist EMG amplitudes of the knee extensors (*r* = −0.3–0.7) and ankle plantar flexors (*r* = −0.4–0.5) (Takai et al., 2008).

The current study also found that participants with weaker back extensors recruited higher antagonist abdominal activation. If muscle recruitment is designed to minimize antagonist activation (Brown and Potvin, 2005) one might question why higher abdominal activation was observed. There are four plausible factors that could explain this finding. First, the line of action of the abdominal oblique sites, specifically the lateral sites have been shown to contribute to generating lateral flexor moments (Brown and Potvin, 2007). These lateral obliques would contribute as an agonist to the lateral flexion moments generated at the beginning and end of the lifting task. Second, the EO1 and IO sites (Arjmand et al., 2008a), balance axial rotation moments produced by unilateral activation of specific back extensor and abdominal muscles (Brown and Potvin, 2007; Arjmand et al., 2008b). These two factors provide a plausible explanation why the oblique sites had higher activation amplitudes than rectus abdominus sites which are designed to produce flexor moments (Table 4) (Brown and Potvin, 2007; Arjmand et al., 2008b). A third factor could be related to the stability demands of the lifting task. These three explanations are related to reduced active system function. The fourth explanation is that those in the WEAK group might have systematic changes in neuromuscular control that result in heightened antagonist co-activation during task performance. This antagonist co-activation may be explained by changes in other spinal systems, and would reduce the net moment produced by a participant. Computational modeling suggests the spine requires antagonist force production to maintain stability (Brown and Potvin, 2005). Whether there is a standard moment that must be produced by the antagonist to ensure joint stability is unknown. If a threshold did exist, weaker individuals would require higher activation amplitudes to meet this threshold. Our data showed that the WEAK group also had significantly lower trunk flexor strength than STRONG (Table 1), supporting that the abdominal muscles compensated for this weakness to fulfill the above-mentioned roles. This finding is consistent with previous work showing individuals with lower knee extensor strength had higher knee flexor EMG activation amplitudes for fundamental tasks such as sit-to-stand and stair ascent/descent (Mizelle et al., 2003).

The results of this study suggest increased EMG activation amplitudes potentially act as a form of adaptation toward reduced force production. While these changes may be necessary to prevent instability events associated with insufficient force production, increases in muscle activation amplitudes represent a unique risk for low back pain. In participants who are WEAK, increased activation amplitudes indicate that muscles are at higher risk to fatigue (van Dieën et al., 2008) resulting in an impaired ability to restore stability through active force (van Dieën et al., 2011). Furthermore, data showing higher agonist and antagonist activation amplitudes suggest that despite the peak net moment being similar between groups (Table 2) altered internal force balance could result in increased spinal compression forces (Granata and Marras, 2000) that can exacerbated joint degeneration (Wang et al., 2007). Going forward more sophisticated models will be required to estimate the moments produced by antagonist muscle groups to support whether this co-activation could lead to higher compressive forces.

Novel to this study was the change in the relative responsiveness of trunk muscles to the dynamic changing moments. For the back extensor sites, WEAK participants were more responsive to changes in the lateral flexion moment produced at the beginning and end of the task (PC2) (Table 3) than STRONG. While not directly measured, sagittal plane trunk strength has been correlated to frontal (*r* = 0.81) and transverse plane (*r* = 0.91) strength (Kocjan and Sarabon, 2014). Thus, weaker individuals should require higher activation of lateral flexor sites to meet the frontal plane moment demands produced by the horizontal transfer task consistent with previous studies showing greater responsiveness (PC2 scores) of trunk muscles sites with increasing external loads (Quirk and Hubley-Kozey, 2014).

### Influence of Recovery From LBP

The secondary purpose of this study was to determine whether the effects of strength on muscle activation patterns were modified by the recent experience of low back pain despite recovery of symptoms. The rLBI reported below clinically meaningful VAS and RMD scores consistent with our inclusion thresholds aimed at assessing recovery. However albeit small, they did have, significantly higher levels of pain (Jensen et al., 2003; Boonstra et al., 2014) and disability (Turner et al., 2004) than the ASYM group on the day of testing (Table 1). Self-reported psychosocial measures were also at the lower end of the pain catastrophizing and fear avoidance scales (Sullivan et al., 1995), however, the statistical analysis captured small but significant differences between the rLBI and ASYM groups (Table 1). Despite mass differences between the rLBI and ASYM group, mass normalized strength (Table 1) and the external moments produced by the horizontal transfer task (Table 2) were similar between groups. As the primary objective of this study was to compare between WEAK and STRONG participants mass deviation between the rLBI and ASYM group would not modify these differences. This was confirmed by including mass as a co-variate in the ANOVA model where there was no change in any group, strength or group by strength interactions captured on PC scores.

The mean strength of participants from this study while within the range of supine trunk flexor strength (136 ± 30 vs. 139.8 ± 35.2 Nm) was above average for prone back extensor strength (212 ± 36 vs. 179.2 ± 31.2 Nm) when compared (vs.) to a cohort of similar 20–60 year old men (Hasue et al., 1980). Higher back extensor strength can partially be explained by the military participants in the current study who have higher levels of fitness (including grip strength) than civilian controls (Deuster et al., 1987), where grip strength has been associated with higher back extensor strength (Wang et al., 2005). In contrast to reports of lower trunk strength in individuals with chronic LBP compared to healthy controls (Hasue et al., 1980; Newton et al., 1993; Steele et al., 2014a), our study found there was no difference between the rLBI and ASYM groups. A result consistent with previous work showing that participants with a history of LBP of varying durations do not have different trunk strength compared to controls (Hultman et al., 1993; McGill et al., 2003a).

Key findings of this study were the two significant strength by group interactions for overall muscle activation amplitudes (PC1) of both the back extensors and abdominals. For the abdominals, the WEAK group had relatively greater differences in overall abdominal activation than STRONG within the rLBI group compared to the ASYM group (Table 3). Comparing ratios for EMG and strength (Table 6), showed that the relative difference in activation amplitudes are likely explained by a greater flexor strength ratio between the STRONG and WEAK rLBI participants. Similarly, for the back extensors, the WEAK rLBI subgroup had higher activation amplitudes than all other subgroups (Table 3). However, unlike in the abdominals contrasting qualitative strength and EMG ratios (Table 6) would suggest this relative difference could not be explained by back extensor strength ratios which were comparable between the STRONG and WEAK ASYM and rLBI subgroups (Table 6).

To account for these EMG differences two theories were investigated. First, previous work in those with chronic LBP suggests that those with higher pain and disability have lower trunk strength (Newton et al., 1993). This link between symptoms and strength may be associated with a reduced ability to produce a true maximum voluntary isometric contraction in those with chronic LBP (Chiou et al., 2013). These differences would inflate the amplitude of MVIC normalized EMG. However, participants in our study were deemed recovered, reporting minimal pain which, has not been shown to limit the ability to produce a true MVIC relative to healthy asymptomatic controls (Chiou et al., 2015). To explore whether pain could explain these differences in EMG amplitudes subjective questionnaires were implemented; however, VAS, RMD, PCS, and TSK were not different between the WEAK vs. STRONG rLBI subgroups (Table 1). Furthermore, to explore whether pain could confound our results we found in the 4 participants with VAS scores characterizing mild pain (ranging from 18 to 28/100 mm) two were in the STRONG and WEAK group, respectively, suggesting the current experience of pain was not a confounder.

A second theory for the higher activation is that the influence of recent pain modified how trunk muscle activation patterns adapt to a stability challenge associated with lower back extensor strength. Experimental work showed, that when an individual is in pain they utilize muscle activation patterns that generally increase the overall activation for all muscle sites to increase joint stiffness and thus stability (Hodges et al., 2013) and according to the motor adaptation to pain theory, once pain resolves some individuals retain these adjusted muscle activation patterns (Hodges et al., 2013). This study expands on this model to hypothesize that individuals with lower active spinal system function (WEAK) might experience greater adaptations to ensure spinal stability that is beyond a margin of safety, whereas those with higher active system function (STRONG) have fewer adjustments. This hypothesis is consistent with recent work showing that changes in trunk muscle activation was greatest when the spine has lower stiffness presenting a higher stability demand (Shojaei et al., 2018). While the results of this study are not definitive, they do support and expand upon the motor adaptation to pain theory. Future research should explore whether those with lower function in a spinal system experience more instability events (painful events). If this were the case it suggests that muscle activation pattern adaptations would remain insufficient and necessitate further adaptations to mitigate the risk of pain. Continued painful events could lead to the appearance of exaggerated adaptation and possibly retention of these pain developed muscle activation patterns. However, more empirical evidence is needed to support the motor adaptation to pain theory and retention of motor patterns post injury.

A limitation of the current study was that only one measure of active spinal system function was assessed; the maximum voluntary isometric moment of the two main muscle groups with no measure of axial or frontal plane muscle strength. Other work has shown isometric trunk strength is correlated between all three fundamental planes (Kocjan and Sarabon, 2014) and hence we chose the back extensor sagittal plane moment as a representative measure of strength that would directly oppose the principal flexion moment produced during this fundamental task. Second, while this study explored whether those with deficits in the active spinal system would have different muscle activation patterns our definition of a deficit was based on participants having below median back extensor strength. While our measure of lower function (WEAK) has been reported as is a potential risk for experiencing future low back pain (Cho et al., 2014), the threshold used in this study was not based on a clinical threshold of deficient function. Encouraging however was that given the potential for overlap in the back extensor strength values between the two groups using the median split approach, there were significant differences in muscle activation patterns with strength and groups. Only including men was a limitation and this was not by study design and not within control. A secondary analysis of only strength main effects (rLBI could not be compared) including the 9 women did not change the key results of this study; however, generalizing the findings from this study to women or non-military populations should be done with caution. A strength of this study was our relatively large sample size with equal numbers between those who have and have not experienced a recent low back injury within the last year. Finally, the study sample included a rLBI group whereas much of our *in vivo* knowledge on low back conditions to date have come from studies focused on those with chronic LBP. Understanding muscle function and adaptations early in the injury process might help in developing clinical targets, where the chronic group findings are often confounded with psychosocial issues and habitual movement patterns.

## Conclusion

Individuals classified with lower back extensor strength utilized different muscle activation patterns to complete a controlled dynamic transfer task than those classified with higher back extensor strength. The results support the hypothesis that participants classified as WEAK adapted muscle activation patterns with higher overall activation amplitudes for both agonist and antagonist sites and in the back extensors only there was greater responsiveness to changing external moments produced in the frontal plane than stronger individuals. These findings not only show that more motor units were recruited to adapt to impaired maximal force production but the relative increase in muscle activation depends on changes in external moment produced by an external task. Secondly, the findings provide evidence that while both an ASYM and rLBI population adapted their muscle activation patterns in a comparable way, having lower strength resulted in greater adaptations in the rLBI group hence recent LBP can alter the relationships between spinal system deficits and trunk muscle activation pattern adaptations.

## Data Availability Statement

All datasets generated for this study are included in the article.

## Ethics Statement

This study was carried out in accordance with the recommendations of the research guidelines from the Research Ethics Board at Dalhousie University. All subjects gave written informed consent in accordance with the Declaration of Helsinki. The protocol was approved by the Research Ethics Board at Dalhousie University (REB# 2016-4059).

## Author Contributions

DQ and CH-K conceived and designed the experiment and analysis. DQ and RT collected the data, where RT ensured quality and accuracy of the clinical assessment and outcomes. DQ performed the analysis and wrote the first draft of the manuscript. All authors discussed the results and contributed to the final manuscript.

### Conflict of Interest

The authors declare that the research was conducted in the absence of any commercial or financial relationships that could be construed as a potential conflict of interest.
